# Tug-of-War Driven by the Structure of Carboxylic Acids: Tuning the Size, Morphology, and Photocatalytic Activity of α-Ag_2_WO_4_

**DOI:** 10.3390/nano12193316

**Published:** 2022-09-23

**Authors:** Lara Kelly Ribeiro, Amanda Fernandes Gouveia, Francisco das Chagas M. Silva, Luís F. G. Noleto, Marcelo Assis, André M. Batista, Laécio S. Cavalcante, Eva Guillamón, Ieda L. V. Rosa, Elson Longo, Juan Andrés, Geraldo E. Luz Júnior

**Affiliations:** 1 Postgraduate Program in Chemistry, Department of Chemistry, Federal University of Piaui, 64049-550, Brazil; 2 LIEC/CDMF, Department of Chemistry, Federal University of São Carlos, P.O. Box 676, 13565-905, Brazil; 3 Department of Physical and Analytical Chemistry, University Jaume I (UJI), 12071 Castellon de La Plana, Spain; 4 Postgraduate Program in Nanoscience in Advanced Materials, Department of Chemistry, Federal University of ABC, 09210-580, Brazil; 5 Postgraduate Program in Chemistry, Department of Chemistry, State University of Piaui, P.O. Box 381, 64002-150, Brazil

**Keywords:** α-Ag_2_WO_4_, sonochemical synthesis, controlled size and morphology, carboxylic acids, photocatalytic activity

## Abstract

Size and morphology control during the synthesis of materials requires a molecular-level understanding of how the addition of surface ligands regulates nucleation and growth. In this work, this control is achieved by using three carboxylic acids (tartaric, benzoic, and citric) during sonochemical syntheses. The presence of carboxylic acids affects the kinetics of the nucleation process, alters the growth rate, and governs the size and morphology. Samples synthesized with citric acid revealed excellent photocatalytic activity for the degradation process of Rhodamine B, and recyclability experiments demonstrate that it retains 91% of its photocatalytic activity after four recycles. Scavenger experiments indicate that both the hydroxyl radical and the hole are key species for the success of the transformation. A reaction pathway is proposed that involves a series of dissolution−hydration–dehydration and precipitation processes, mediated by the complexation of Ag^+^. We believe these studies contribute to a fundamental understanding of the crystallization process and provide guidance as to how carboxylic acids can influence the synthesis of materials with controlled size and morphology, which is promising for multiple other scientific fields, such as sensor and catalysis fields.

## 1. Introduction

α-Ag_2_WO_4_ is a representative member of Ag-based complex oxides and, due to its unique properties, is employed in a wide range of applications such as catalysts, photocatalysts, electrocatalysts, photoluminescent, sensors, adsorbents of dyes, and antimicrobial and bactericidal agents [1,2,3,4]. α-Ag_2_WO_4_ has been prepared by using different methods, such as simple precipitation [5], controlled precipitation (CP) [6], chemical precipitation [7], coprecipitation at different temperatures [8], coprecipitation followed by microwave irradiation [9], conventional hydrothermal methods [10,11], microwave-assisted hydrothermal (MAH) methods [12], simple electrochemical methods [13], simple ion exchanges at room temperature [14], and sonochemical (SC) methods [15].

The size and morphology of crystals govern their properties for a range of important applications. These characteristics are thermodynamically controlled by the values of surface energies, allowing a crystal with lower-energy surfaces to achieve more stability [16]. The realization of crystals with a defined size and morphology requires the efficient control of nucleation and growth processes, including not only the precise adjustment of the decisive reaction parameters (e.g., concentrations and temperature) but also the use of selected surface-active agents to block or to support the growth of specific crystal surfaces [17]. In this regard, surface ligands play a pivotal role in obtaining crystals with a defined size and regulated morphology and function during the synthesis via the judicious control of reaction conditions [18,19]. This ability offers unique and tunable properties, and typical examples of surface ligands are carboxylic acids (CAAs). They are chelating agents and react with metal cations to form stable metal complexes, thereby preventing their agglomeration, and they play multiple roles from the regulation of the solubility and availability of active components during syntheses to their capability to stabilize different surfaces of crystals [20,21,22]. Understanding the underlying mechanism for the growth of a material greatly assists control over its synthesis. However, the complexity of crystal surfaces makes it challenging to determine the local environment of active sites and the role of ligands in controlling the properties and activity.

Our group is engaged in a research project devoted to controlling the size and morphology of α-Ag_2_WO_4_ by selecting different ligands [23]. Here, the SC method is employed for the synthesis of α-Ag_2_WO_4_ using different CAAs (tartaric (TA), benzoic (BA), and citric (CA)). The challenge is two-fold: the first challenge is to establish a comprehensive understanding of the relationship between the size, morphology, and photocatalytic activity for the degradation of Rhodamine B (RhB) using different CAAs; the second challenge is to clarify how their presence affects the kinetics of nucleation and growth processes. The samples were characterized by X-ray diffraction (XRD) and X-ray photoelectron spectroscopy (XPS), Raman and Fourier-transform infrared (FTIR) spectroscopies, field-emission scanning electron microscope (FE-SEM), and transmission electron microscopy (TEM) images. The optical properties of the α-Ag_2_WO_4_ samples were investigated using measurements from ultraviolet-visible (UV-Vis) spectroscopy.

## 2. Materials and Methods

### 2.1. Synthesis

Silver nitrate (2 × 10^−3^ mol)(AgNO_3_, 99.99% purity, Sigma Aldrich, (St. Louis, MI, USA), sodium tungstate dihydrate (1 × 10^−3^ mol)(NaWO_4_∙2H_2_O, 99.99% purity, Sigma Aldrich, (St. Louis, MI, USA), CAAs (8 × 10^−3^ g): tartaric acid (C_4_H_6_O_6_, 99.00% purity, Sigma Aldrich, (St. Louis, MI, USA), benzoic acid (C_7_H_6_O_2_, 99.00% purity, Sigma Aldrich, (St. Louis, MI, USA), and citric acid (C_6_H_8_O_7_, 99.00% purity, Sigma Aldrich, (St. Louis, MI, USA) were used as precursors.

**Figure 1:**
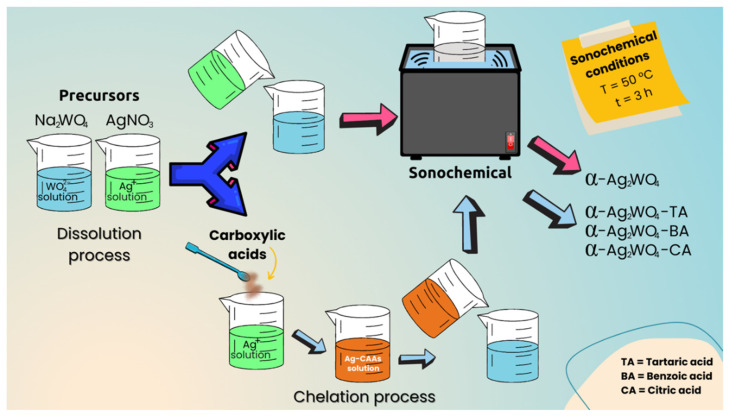
Illustration of the synthesis process of the α-Ag_2_WO_4_, α-Ag_2_WO_4_-TA, α-Ag_2_WO_4_-BA, and α-Ag_2_WO_4_-CA samples.

Solutions of AgNO_3_ and NaWO_4_·2H_2_O salts were prepared separately in 200 mL of deionized water at 25 °C, and the corresponding CAA was added to the AgNO_3_ solution. Then, both solutions were mixed and transferred to the ultrasound (Branson Ultrasonic Cleaner model CPX-1800h, (Emerson Electric Co, Bethel, USA) at a frequency of 40 kHz for 3 h. Afterward, pH values of each solution were measured, obtaining 7.62, 6.72, 6.53, and 7.11 without CAA, TA, BA, and CA, respectively. Finally, the samples were washed (×10) with deionized water by centrifugation to remove residual Na^+^ ions and organic residues. The resulting solid was collected and dried at 65 °C for 10 h. A scheme of the synthetic route is illustrated in Figure 1. α-Ag_2_WO_4_, α-Ag_2_WO_4_-TA, α-Ag_2_WO_4_-BA, and α-Ag_2_WO_4_-CA are used to designate samples synthesized without CAA, and those synthesized with TA, BA, and CA, respectively.

### 2.2. Characterization

Details about the characterization techniques are presented in the Supplementary Materials (see section SM-1).

### 2.3. Photocatalytic Activity Measurements

#### 2.3.1. Photodegradation

The photocatalytic activity of the samples was evaluated through the photocatalytic degradation of 50 mL of RhB (P.A., Synth) in an aqueous solution under UV-Vis light. In a typical process, 50 mg of the synthesized materials was dispersed in 50 mL of the RhB solution (1 × 10^−5^ mol L^−1^) for 10 min in an ultrasonic bath (42 kHz, model 1510). The mixture was then transferred to a 100 ml glass bottle and stirred for 30 min in the dark for the homogeneous dispersion of the catalyst and to allow adsorptive processes. Then, the suspensions were irradiated with six UV lamps (PHILIPS TL-D, São Paulo, BR, 15 W) at a distance of 10 cm from the reactor under vigorous stirring, and the temperature was maintained at 20 °C via a thermostatic bath. At predetermined times (0, 10, 20, 30, 40, 60, and 90 min), a 2 mL aliquot of the suspension was removed from the photocatalytic system and placed into a plastic tube. Afterward, the suspension was centrifuged at 10,000 rpm for 5 min for the complete removal of the catalyst particles. The remaining solution was analyzed by UV-Vis absorption spectroscopy on a V-660 spectrophotometer (JASCO) in order to monitor the variations in the absorption band of RhB, with the maximum at λ = 554 nm for all photocatalytic tests.

#### 2.3.2. Photocatalytic Concentration and Photodegradation Rate

The effect of the photocatalytic concentration on the photodegradation rate was analyzed by using the following ratios: 0.5 mg/mL (25 mg of catalyst in 50 mL of RhB), 1 mg/mL (50 mg of catalyst in 50 mL of RhB), 2 mg/mL (100 mg of catalyst in 50 mL of RhB), and 4 mg/mL (200 mg of catalyst in 50 mL of RhB). The procedure adopted was the same as described in Section 2.3.1.

#### 2.3.3. Scavenger Measurements

The identification of the reactive oxygen species (ROS) was performed by scavenger tests. For this purpose, equivalent amounts of benzoquinone (BQ, Merck, Darmstadt, DE), ammonium oxalate (AO, 99.5%, Sigma Aldrich, St. Louis, MI, USA), AgNO_3_ (99.8%, Vetec, RJ, BR), and tert-butyl alcohol (TBA, 99%, Sigma Aldrich, St. Louis, MI, USA), were added to reaction vials containing 50.0 mL of the RhB solution (1 × 10^−5^ mol L^−1^) to identify •O_2_^−^, h^+^, e^−^, and •OH, species, respectively.

## 3. Results

An analysis of XRD data (see Figure S1) renders that all samples have well-defined diffraction peaks, indicating a good degree of structural order. The as-synthesized α-Ag_2_WO_4_ samples present an orthorhombic structure belonging to the symmetry Pn2n space group, according to card No. 4165 in the Inorganic Crystal Structure Database (ICSD), showing that the SC method proved to be efficient for the synthesis of α-Ag_2_WO_4_ materials. It is verified in Figure S1 that the gradual increase in the full width at maximum (FWHM) of the plane (321) for the CAAS samples when compared to the pure α-Ag_2_WO_4_ sample is related to the reduction in crystallite sizes. The values of the lattice parameters, unit cell volume, and statistical parameters of quality obtained by Rietveld refinements are presented in Table S1. According to the statistical parameters obtained in the Rietveld refinement in Table S1, the quality of the structural refinement data is acceptable.

Raman and FTIR spectroscopy were also used to characterize all samples. From the Raman spectra of the α-Ag_2_WO_4_, α-Ag_2_WO_4_-TA, α-Ag_2_WO_4_-BA, and α-Ag_2_WO_4_-CA samples in Figure S3A, it is possible to observe that the active modes between 500 and 100 cm^−1^ are related to external vibrational modes of [AgO*_x_*] (*x* = 2, 4, 6, and 7). The active modes between 500 and 1000 cm^−1^ can be attributed to vibration motions in the atoms of the [WO_6_] clusters. Among them, the intense band at 878 cm^−1^ is assigned to the symmetrical stretching of the W−O bond in octahedral [WO_6_] clusters. As a complementary analysis to Raman spectroscopy, FT-IR measurements were performed. Figure S3B Illustrated the FT-IR spectra and the corresponding positions of IR-active modes of α-Ag_2_WO_4_ samples. The tungstate with a scheelite-type structure has eight stretching and/or bending IR-active vibrational modes [24]; however, only two were identified in the α-Ag_2_WO_4_ samples between the spectral range of 400 and 900 cm^−^^1^. These modes are located at 802 and 849 cm^−^^1^ and can be attributed to the overlapping of two intense bands, referring to A_u_ and E_u_, respectively, whereas the IR-active modes are ascribed to the O−W−O anti-symmetric stretching vibrations in the [WO_6_] clusters.

XPS measurements identify the elemental composition, oxidation state, the overall electronic structure, and the density of the electronic states in the material (see Figure S4A–D). There were no expected percentages of C 1s in the samples, and the large amount of C 1s observed in the α-Ag_2_WO_4_-CA sample was assigned to the sample holder once the samples were dried for 10 h at 60 °C. The characteristic peaks of the Ag, W, and O atoms indicate a high purity for all samples. The binding energy values calculated for all samples agree with those in the literature for α-Ag_2_WO_4_ samples [25,26].

High-resolution XPS spectra were performed for the elements forming the α-Ag_2_WO_4_ structure. The spectra of the Ag species in Figure S5A show two bands located between ∼368 and ∼374 eV, which can be attributed to the binding energies of Ag 3*d*5/2 and 3*d*3/2, respectively, while the XPS spectra of the W species in Figure S5B show two bands located between ∼36 and ∼34 eV, which can be attributed to W 4*f*_7/2_ and 4*f*_5/2_ binding energies, respectively, and a broad peak related to W 5*p*_3/2_ is located between 40.7 and 41.2 eV [3]. More information about the XPS spectra of Ag and W species can be found in Figure S5.

**Figure 2:**
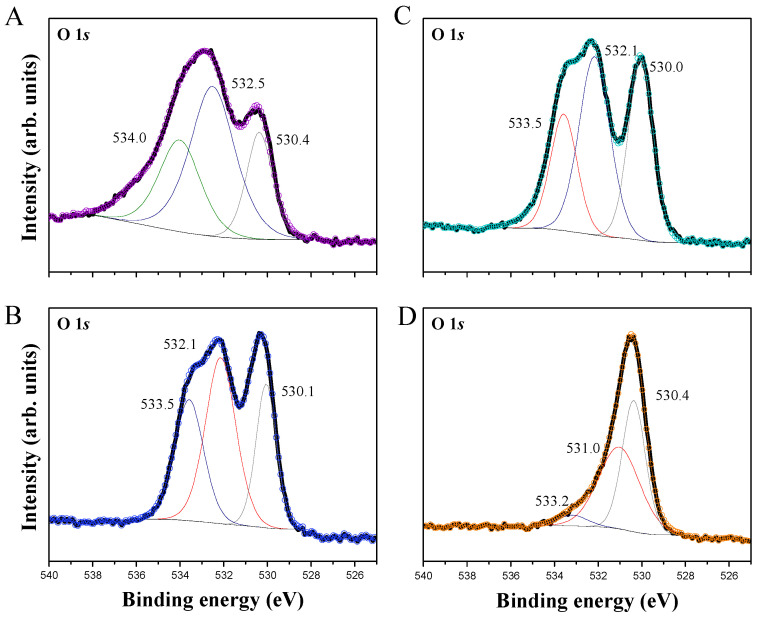
High-resolution XPS spectra of the O 1s orbital of α-Ag_2_WO_4_ (**A**), α-Ag_2_WO_4_-TA (**B**), α-Ag_2_WO_4_-BA (**C**), and α-Ag_2_WO_4_-CA (**D**) samples.

High-resolution XPS spectra of O *1s* atoms present in α-Ag_2_WO_4_, α-Ag_2_WO_4_-TA, α-Ag_2_WO_4_-BA, and α-Ag_2_WO_4_-CA samples are illustrated in Figure 2. These XPS spectra were deconvoluted into three binding energy (BE) values corresponding to lattice oxygens (L_O_), oxygen vacancies (V_O_), and surface hydroxyl/water or chemisorbed oxygen species (S_O_), with BE values ranging from 530.0 to 530.4 eV, 532.0 to 532.5 eV, and 533.2 to 534.0 eV, respectively. The presence of L_O_, V_O_, and S_O_ is responsible for the asymmetric nature of the observed energy peak in O 1*s* spectra. Changes in the BE values provoke shifts in the XPS spectra due to being associated with the amount of the three different types of oxygen. Based on this relationship in Table 1, the percentages of L_O_, V_O_, and S_O_ for all samples are presented. There is an increase in the area percentage of the L_O_ from 18.35% (α-Ag_2_WO_4_) to 57.68% (α-Ag_2_WO_4_-CA), which induces a decrease in the amount of the other two types of oxygen, V_O_ and S_O_.

The results of the UV-Vis diffuse reflectance spectra of the α-Ag_2_WO_4_ samples are shown in Figure S6A–D, and the values of the *E*_gap_ follow this order: α-Ag_2_WO_4_ > α-Ag_2_WO_4_-TA > α-Ag_2_WO_4_-BA > α-Ag_2_WO_4_-CA. From these results, we can propose that in the α-Ag_2_WO_4_-CA, there is a formation of intermediate levels in the band gap region that can be associated with the increase in the amount of L_O_.

**Figure 3:**
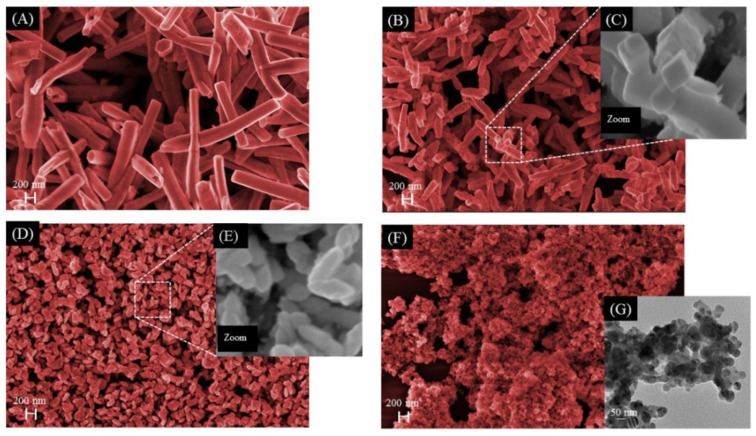
FE-SEM images of α-Ag_2_WO_4_ (**A**), α-Ag_2_WO_4_-TA (**B**) (inset (**C**), images of the formation of rectangular morphology), α- Ag_2_WO_4_-BA (**D**) (inset (**E**), formation of nanometric rice-grains), and α-Ag_2_WO_4_-CA (**F**). TEM images for α-Ag_2_WO_4_-CA showing higher magnifications of the nanoparticles (**G**).

A detailed analysis of the images from the FE-SEM and TEM techniques in Figure 3 reveals that the different CAAs provoke changes in the morphology of the as-synthetized samples. The characteristic morphology of α-Ag_2_WO_4_, already reported in the literature, corresponds to long prisms or needles with bases similar to a hexagon, composed of the (010), (001), and (101) exposed surfaces [23,27,28], as can be seen in Figure 3A. The images represented in Figure 3B,C show a rectangular morphology for the α-Ag_2_WO_4_-TA sample due to the stabilization of the (100) surface with respect to the (101) surface [23]. The image of the α-Ag_2_WO_4_-BA sample (Figure 3D) shows a change in the α-Ag_2_WO_4_ morphology from well-defined surfaces to rice-grains with poorly defined surfaces (see Figure 3E). A dramatic particle size reduction is sensed in the α-Ag_2_WO_4_-CA sample (Figure 3F).

**Figure 4:**
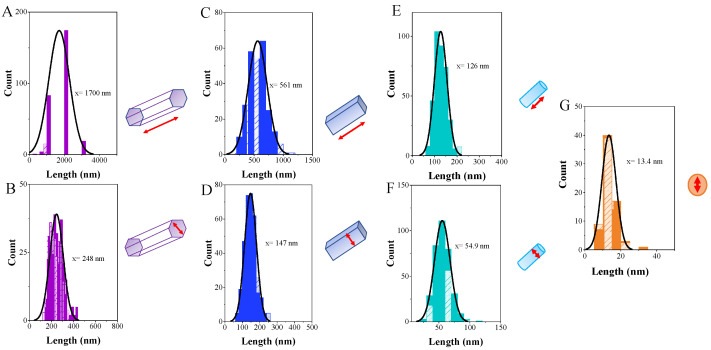
Average length and width distribution of α-Ag_2_WO_4_ (**A**,**B**), α- Ag_2_WO_4_-TA (**C**,**D**), and α- Ag_2_WO_4_-BA (**E**,**F**) via FE-SEM. Average length and width distribution of α-Ag_2_WO_4_-CA (**G**) via TEM images.

The average distribution of length and width of crystallites is reported in Figure 4, and it was obtained from the FE-SEM and TEM images (Figure 3) using the program GNU Image Manipulation Program. An analysis of the results in Figure 4 renders that the average values of length and width, respectively, have decreased as follows: 1700 nm and 248 nm for α-Ag_2_WO_4_ (Figure 4A,B), 561 nm and 147 nm for α-Ag_2_WO_4_-TA (Figure 4C,D), and 126 nm and 54.9 nm for α-Ag_2_WO_4_-BA (Figure 4E,F). For the α-Ag_2_WO_4_-CA sample, a spheroidal morphology was observed by TEM, and there is no distinction between length and width, with an average size value of 13.4 nm (Figure 4G). It is important to remark that this is the first time this morphology for α-Ag_2_WO_4_ has been reported. In addition, we also calculated the size of the α-Ag_2_WO_4_-CA crystal by using the Scherrer equation and the Halder–Wagner–Langford method [29,30], and values of 13.33 and 16.84 nm have been obtained, respectively. These three values are very similar.

The decrease in particle size with the addition of CAAs can be explained as a result of the formation of a chelate complex with Ag^+^ cations. Ion dissociation and pairing are ubiquitous phenomena occurring in dissolution processes. Therefore, in the first step, H_2_O molecules mainly dissociate AgNO_3_ to solvate Ag^+^ cations to form [Ag(H_2_O)_2_]^+^∙nH_2_O [31]. In the next stage, the presence of CAAs, in turn, causes H_2_O molecules to gradually become a relatively worse partner of the Ag^+^ cation due to the fact that a more stable bond with carboxylic groups (COO^−^) can be formed. Thus, H_2_O molecules play an indirect role in weakening the Ag^+^ hydration shell via the dehydration process, which may be interpreted as the beginning of the chelation processes of Ag^+^ with the different CAAs to form a strong bond with COO^−^ moieties.

**Figure 5:**
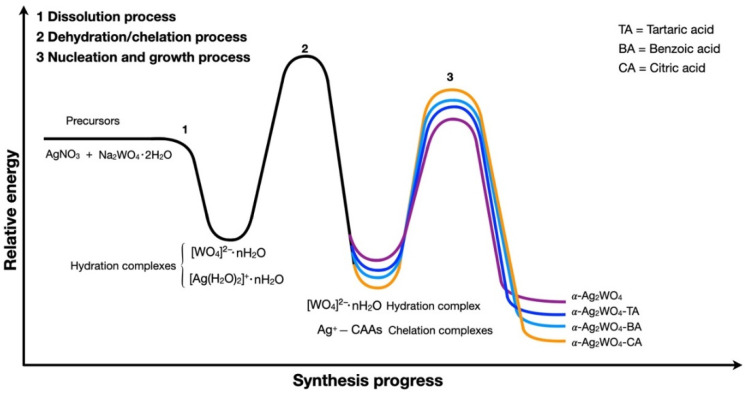
Schematic representation of the energy profiles for the synthesis progress.

It is expected that the nucleation and growth processes of α-Ag_2_WO_4_ are controlled by the strong binding effect of CAAs with Ag^+^ cations because both processes are directly related to the release kinetics for the formation of Ag^+^ cations. The strong binding effect of CAAs prevents the agglomeration of Ag^+^ cations. Based on the above considerations, a schematic representation of the synthesis progress is proposed, which involves a series of dissolution, dehydration, chelation, nucleation, and growth processes, mediated by the complexation of Ag^+^, as shown in Figure 5.

The tug-of-war between the formation of the [Ag (H_2_O)_2_]^+^ nH_2_O complex and the chelation process controls the release of Ag^+^ as the synthesis progresses. There is a dynamic balance between the strengths of the Ag−O and Ag−CAAs bonds in the hydration and chelated complexes, respectively. Therefore, the presence of CAAs serves as a template directing the size and morphology of the as-synthesized samples. The stabilization of the chelate complex is directly linked to the nucleation and growth process of α-Ag_2_WO_4_; thus, in this case, it is observed that the lower the stability of the formed chelate complex, the larger the average size of the obtained samples. The experimental results further support the proposed mechanism; the molecular-level interactions involving Ag^+^ cations in H_2_O and CAAs drive the size and morphology of the as-synthesizer samples.

**Figure 6:**
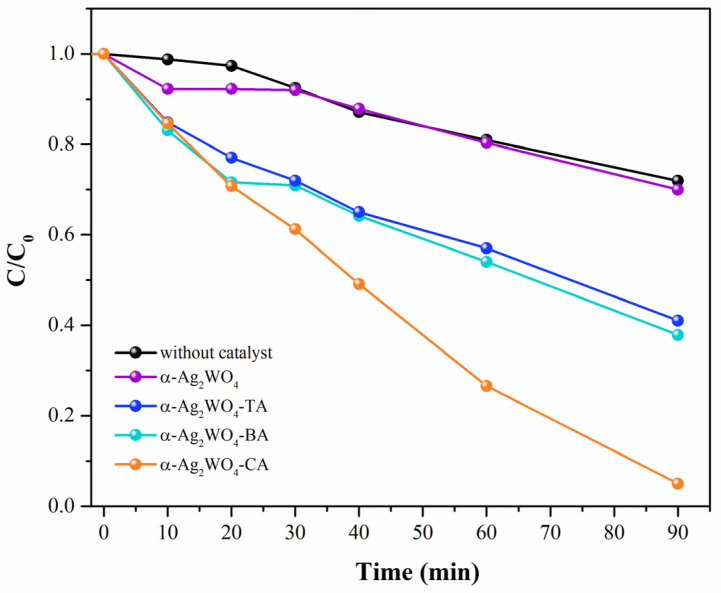
The variation of RhB concentration (C/C_0_) as a function of irradiation time.

The photocatalytic performance was investigated via the degradation of RhB. The time-dependent curve of the concentration and spectrum during RhB degradation is shown in Figure 6 and Figure S7, respectively.

The degradation process follows first-order kinetics and can be described by d[C]/dt=k[C], where [C] is the RhB concentration and k indicates the rate constant, which can be obtained from the graphical representation of the integrated equation (see Figure 6). The corresponding rate constant value for the RhB degradation was 4.13 × 10^−3^ min^−1^, while in the presence of the α-Ag_2_WO_4_, α-Ag_2_WO_4_-TA, α-Ag_2_WO_4_-BA, and α-Ag_2_WO_4_-CA catalysts, the values were 3.32 × 10^−3^ min^−1^, 8.86 × 10^−3^ min^−1^, 9.43 × 10^−3^ min^−1^, and 3.46 × 10^−2^ min^−1^, respectively. These results show that the presence of CAAs in the SC synthesis increases the degradation process of RhB dye, as demonstrated in Table 2 when the activity is compared to those reported in the literature [9,23,32,33] under the same lamp (UV-Vis). The α-Ag_2_WO_4_-CA catalyst exhibited excellent catalytic performance (95% of degradation in 90 min) due to its optimal size effect with a higher surface/volume ratio and the presence of plenty of active sites at the sphere-like morphology.

To analyze the effect of the amount of α-Ag_2_WO_4_-CA catalyst on the photodegradation rate, the photocatalyst’s concentration was increased to 2 and 4 mg/mL. For both cases, 100% of degradation was achieved in 60 min, and the rate constant values were 68.25 and 55.81 × 10^−3^ min^−1^, respectively (Figure S8).

**Figure 7:**
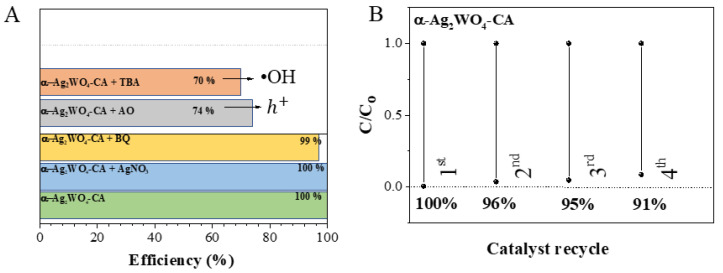
Photocatalytic RhB degradation profiles using α-Ag_2_WO_4_-CA in the presence of different scavengers (**A**) and reuse of the α-Ag_2_WO_4_-CA catalyst after catalytic cycles (**B**).

To investigate the active species along the photodegradation mechanism, the trapping experiments were performed only for the best photocatalytic (α-Ag_2_WO_4_-CA). To try to correlate the size/morphology/photocatalytic activity and the nature of the ROS for all samples, it was necessary to perform a trapping experiment with α-Ag_2_WO_4_, α-Ag_2_WO_4_-TA, and α-Ag_2_WO_4_-BA samples. However, this is out of the scope of our work. As shown in Figure 7A, the addition of TBA and AO had an obvious effect, which revealed that both •OH and $h^{+}$ were the primary active substance in the photocatalytic process. However, after adding BQ and AgNO_3_, no changes are sensed, which means that •O_2_^−^ and e^−^ had little effect on photocatalytic reactions.

Reuse experiments using 2 mg/mL were performed to evaluate the photocatalytic stability of the α-Ag_2_WO_4_-CA. The results are shown in Figure 7B, where it is possible to observe that the sample decreases in photocatalytic activity at each cycle, decomposing 91% of the dye in the fourth photocatalytic cycle, thus demonstrating that the material has stability. In Figure S9, the loss of mass of the α-Ag_2_WO_4_-CA along the catalytic cycles is presented. An analysis of the results reveals that a low decrease in photocatalytic efficiency can be associated with the loss of mass after the fourth cycle.

**Figure 8:**
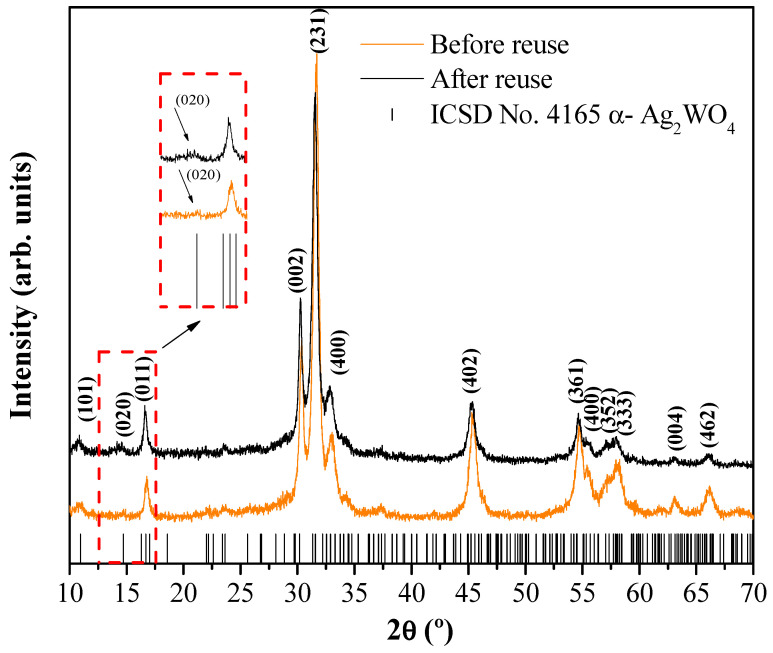
XRD of α-Ag_2_WO_4_-CA before and after its use in the degradation of RhB. The vertical lines indicate the respective positions of α-Ag_2_WO_4_ found on the ICSD card 4165.

Structural characterization for assessing the stability of α-Ag_2_WO_4_-CA after the fourth cycle was performed. In Figure 8, the XRDs before and after reuse are presented. An analysis and comparison of the results show that the positions of the different peaks are similar.

## 4. Conclusions

Crystal size and morphology engineering of metal oxides is a promising route for tuning their properties and enhancing their performance. These characteristics depend on preparation conditions, which in turn can also affect the surface chemistry and reactivity. Therefore, it is critical to have tight control over their size and morphology as these parameters have strong correlations with a range of properties. Size- and morphology-controlled synthesis can be performed by using surface ligands, such as surfactants, due to their capability to stabilize different exposed surfaces at different morphologies. This work presents a facile synthesis strategy for altering the morphologies and sizes of α-Ag_2_WO_4_ by using three carboxylic acids (tartaric, benzoic, and citric) as chelating agents, thereby modulating their photocatalytic activity. The main conclusions of the present work can be summarized as follows: (i) a comprehensive understanding of the relationship between the size, morphology, and photocatalytic activity is provided; (ii) plausible mechanisms to explain the kinetics of nucleation and growth processes are proposed; (iii) a tug-of-war between dehydration and chelation processes is disclosed to control the rate of Ag^+^ release for tuning the size, morphology, and photocatalytic activity of the as-synthetized α-Ag_2_WO_4_ material; (iv) the α-Ag_2_WO_4_ samples synthesized with citric acid have nanoscale dimensions and reached 100% degradation of RhB in 60 min at the concentration of 2 mg/mL; (v) hydroxyl radical, •OH, and hole, $h^{+}$, are the main oxidative species, as indirectly evidenced by means of scavenging experiments; (vi) recycling tests render that α-Ag_2_WO_4_-CA nanomaterials are stable after four cycles. Finally, we hope that the present findings and concepts can be applied to future research directions for the controlled synthesis of complex metal oxides with desirable size and morphology. Subsequent research will investigate the influence of the crystal structure on their properties and provide opportunities for further development.

## Figures and Tables

**Table 1:** Calculated percentage values of the amount of L_O_, V_O_, and S_O_ (%) based on the deconvolution of the O 1*s* peaks as a function of BE.

Samples	L_O_	V_O_	S_O_
α-Ag_2_WO_4_	18.35	49.63	32.05
α-Ag_2_WO_4_-TA	33.82	47.44	18.74
α-Ag_2_WO_4_-BA	35.86	28.79	35.61
α-Ag_2_WO_4_-CA	57.68	28.39	13.93

**Table 2:** Comparative results for the RhB degradation in the presence of α-Ag_2_WO_4_ materials.

Synthesis	Size of Particles (nm)	E_gap_ (eV)	Photocatalyst Concentration (mg/mL)	Dye Removal (%)	Time (min)	First-Order Kinetics × 10^−3^ (min^−1^)	Ref.
Hydrothermal	200–600	--	1.0	100	60	---	[33]
CP with SDS **	33	3.20	1.0	37	120	1.6	[23]
MAH	600	--	1.0	100	210	6.84	[9]
SC	248	3.03	1.0	30	90	3.32	This work
SC with TA	147	2.94	1.0	58	90	9.43	This work
SC with BA	54.9	2.79	1.0	62	90	8.86	This work
SC with CA	13.4	2.71	1.0	95	90	34.96	This work

SDBS = sodium dodecylbenzene sulfonate. ** SDS = sodium dodecyl sulfonate.

## Data Availability

Not applicable.

## Supplementary Materials

The following supporting information can be downloaded at: https://www.mdpi.com/article/10.3390/nano12193316/s1, Figure S1. XRD patterns of α-Ag_2_WO_4_, α-Ag_2_WO_4_-TA, α-Ag_2_WO_4_-BA, and α-Ag_2_WO_4_-CA samples. The vertical lines indicate the respective positions found on the α-Ag_2_WO_4_ ICSD card 4165; Figure S2. Rietveld refinements of the α-Ag_2_WO_4_, α-Ag_2_WO_4_-TA, α-Ag_2_WO_4_-BA, and α-Ag_2_WO_4_-CA samples; Table S1. Lattice parameters (a, b, c) and unit cell volume (V) with their standard deviation and statistical parameters of quality obtained by Rietveld refinement for α-Ag_2_WO_4_, α-Ag_2_WO_4_-TA, α-Ag_2_WO_4_-BA, and α-Ag_2_WO_4_-CA samples. R_wp_: weighted profile factor, R_p_: profile factor, R_Bragg_: Bragg factor, χ^2^: reduced chi-square. Figure S3. Raman spectroscopy of samples. The vertical dashed lines indicate the position of the Raman peaks and active modes (A). FTIR spectra of samples. The vertical lines indicate the relative positions of the infrared-active modes (B); Figure S4. XPS spectra of the samples of α-Ag_2_WO_4_, α-Ag_2_WO_4_-TA, α-Ag_2_WO_4_-BA, and α-Ag_2_WO_4_-CA; Figure S5. High-resolution XPS spectra of Ag 3d (A, B, C, and D) and W 4f (E, F, G, and H) orbitals; Figure S6. Determination of the *E*_gap_ values. Plots of transformed Kubelka – Munk function versus photon energy for α-Ag_2_WO_4_ (A), α-Ag_2_WO_4_-TA (B), α-Ag_2_WO_4_-BA (C), α-Ag_2_WO_4_-CA (D) samples; Figure S7. Evolution of UV−vis absorption spectra after 90 min of illumination for the degradation of RhB by the α-Ag_2_WO_4_ (A), α-Ag_2_WO_4_-TA (B), α-Ag_2_WO_4_-BA (C), and α-Ag_2_WO_4_-CA (D) samples; Figure S8. Photocatalytic RhB degradation profiles using the α-Ag_2_WO_4_-CA at different concentrations; Figure S9. Relationship between loss of mass of the α-Ag_2_WO_4_-CA and degradation efficiency during the catalytic cycles.
