# Supplementary material for: Tug-of-War Driven by the Structure of Carboxylic Acids: Tuning the Size, Morphology, and Photocatalytic Activity of α-Ag2WO4
Source: Nanomaterials (Basel). 2022 Sep 23;12(19):3316. doi: 10.3390/nano12193316 (PMC9565223; doi:10.3390/nano12193316)
Supplement: Supplementary file 1 [file nanomaterials-12-03316-s001.zip › nanomaterials-1860388-supplementary.pdf]

Supplementary Material

Tug-of-War Driven by the Structure of Carboxylic Acids: Tuning the Size, Morphology, and Photocatalytic Activity of α-Ag_2WO_4

Lara Kelly Ribeiro [1,2,3,†], Amanda Fernandes Gouveia [3,†], Francisco das Chagas M. Silva [1], Luís F. G. Noleto [1], Marcelo Assis [2,3], André M. Batista [4], Laécio S. Cavalcante [5], Eva Guillamón [3], Ieda L. V. Rosa [2], Elson Longo [2], Juan Andrés [3,*] and Geraldo E. Luz Júnior [1,4]

[1] Postgraduate Program in Chemistry, Department of Chemistry, Federal University of Piaui, 64049-550, Brazil
[2] LIEC/CDMF, Department of Chemistry, Federal University of São Carlos, P.O. Box 676, 13565-905, Brazil
[3] Department of Physical and Analytical Chemistry, University Jaume I (UJI), 12071 Castellon de La Plana, Spain
[4] Postgraduate Program in Nanoscience in Advanced Materials, Department of Chemistry, Federal University of ABC, 09210-580, Brazil
[5] Postgraduate Program in Chemistry, Department of Chemistry, State University of Piaui, P.O. Box 381, 64002-150, Brazil
* Correspondence: andres@qfa.uji.es; Tel.: +34-669-36-94-11
† The authors contributed equally to this work.

SM-1 Characterization

The crystalline phase of the samples was evaluated by X-ray diffraction (XRD) using a D/Max-2500PC diffractometer (Rigaku, Japan) with Cu Kα radiation (λ = 0.154184 nm), at a diffraction angle 2θ ranging from 10° to 110° and a scanning step of 0.02 °/min. The experimental lattice parameters, unit cell volumes and atomic positions were calculated with the aid of GSAS program. For the analysis of the atomic compositions, X-ray photoelectron spectroscopy (XPS) was performed on a Thermo Fischer Scientific spectrometer model K-alpha+. Monochromatic Al Kα radiation was used as an excitation source. Energy steps of 0.5 and 0.05 eV were used for survey and high-resolution spectra, respectively. The binding energies in all spectra were calibrated in reference to the C 1*s* peak (284.8 eV). The W 4*f*, Ag 3*d*, and O 1*s* core levels were measured in high-resolution mode. CasaXPS1 software was used to analyze the XPS spectra, in which the core-level signals were individually fitted with Gaussian-Lorentzian functions and background subtraction according to the Shirley method. Measurements of micro-Raman (MR) spectrum were collected on a T6400 spectrometer (HoribaJobin-Yvon) coupled to a CCD Synapse detector equipped with an argon-ion laser of 514 nm operating at 7 mW. These spectra were obtained over wavenumbers ranging from 50 cm^{-1} to 1100 cm^{-1}. Fourier-transform infrared spectroscopy (FTIR) was performed using a Jasco FT/IR-6200 spectrophotometer (Japan) operating in absorbance mode at room temperature. The spectra were collected in the range of 200–900 cm^{-1}. The optical properties of the samples were investigated through measurements of ultraviolet-visible (UV-Vis) spectroscopy. The value of optical energy band gap (E_{gap}) was calculated using the Kubelka and Munk-Aussig method from transmittance measurements taken on a Shimadzu spectrophotometer (model UV2600). The shapes and sizes of the samples were observed with a field emission scanning electron microscope (FE-SEM) operating at 5–10 kV (Supra 35-VP, Carl Zeiss). Transmission electron microscopy (TEM) images were obtained on a Jeol JEM-2100F operating at 200 kV.

SM-2 Diffractograms of the α-Ag_2WO_4 samples

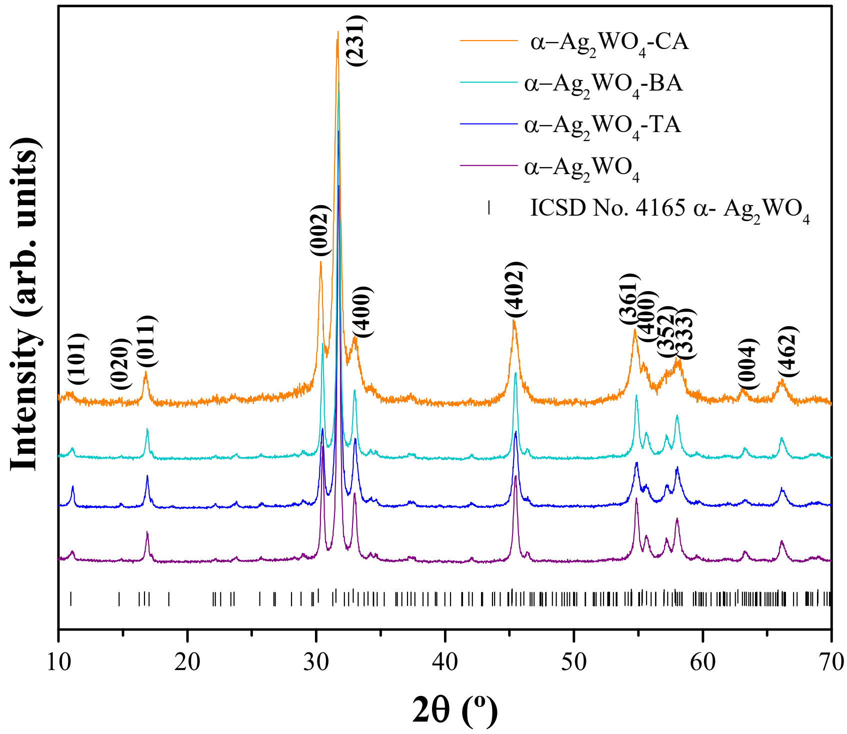

Figure S1. XRD patterns of α-Ag_2WO_4, α-Ag_2WO_4-TA, α-Ag_2WO_4-BA, and α-Ag_2WO_4-CA samples. The vertical lines indicate the respective positions found on the α-Ag_2WO_4 ICSD card 4165.

SM-3 Rietveld refinement plot of α-Ag_2WO_4 samples

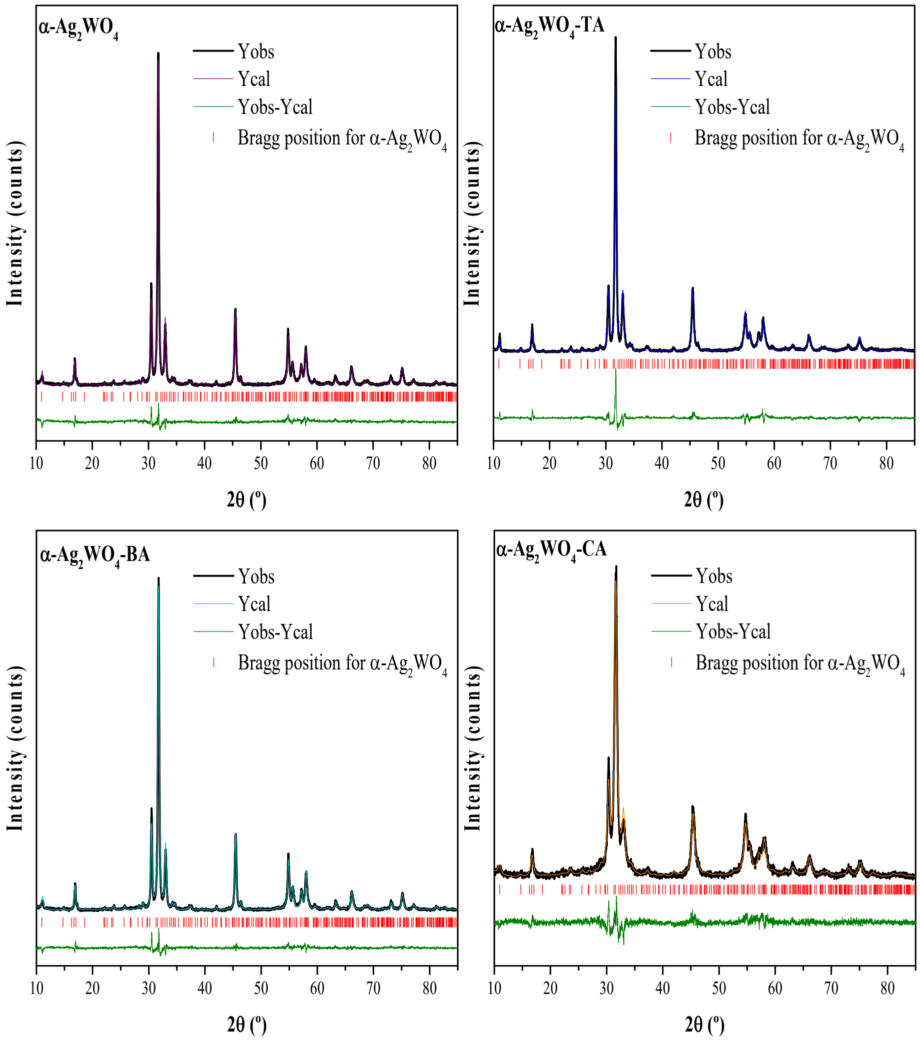

Figure S2. Rietveld refinements of the α-Ag_2WO_4, α-Ag_2WO_4-TA, α-Ag_2WO_4-BA, and α-Ag_2WO_4-CA samples.

Table S1. Lattice parameters (a, b, c) and unit cell volume (V) with their standard deviation, and statistical parameters of quality obtained by Rietveld refinement for α-Ag_2WO_4, α-Ag_2WO_4-TA, α-Ag_2WO_4-BA, and α-Ag_2WO_4-CA samples. R_{wp}: weighted profile factor, R_p: profile factor, R_{Bragg}: Bragg factor, χ^2: reduced chi-square.

Samples	a (Å)	b (Å)	c (Å)	V (Å^3)	R_{wp} (%)	R_p (%)	R_{Bragg} (%)	χ^2
α-Ag_2WO_4	10.90346 (43)	12.01760 (54)	5.88866 (22)	771.611 (33)	11.10	8.41	6.78	2.249
α-Ag_2WO_4-TA	10.90170 (38)	12.01563 (48)	5.88766 (19)	771.229 (29)	9.80	7.49	8.45	1.755

α-Ag_2WO_4-BA	10.89220 (62)	12.02236 (78)	5.89549 (32)	772.014 (49)	14.56	11.33	5.98	2.686
α-Ag_2WO_4-CA	10.86179 (48)	12.02843 (15)	5.89618 (83)	770.337 (71)	12.08	9.40	7.64	1.716
ICSD-4165	10.892	12.032	5.922	775.560	–	–	–	–

SM-4 Raman and FTIR spectroscopy of α-Ag_2WO_4 samples

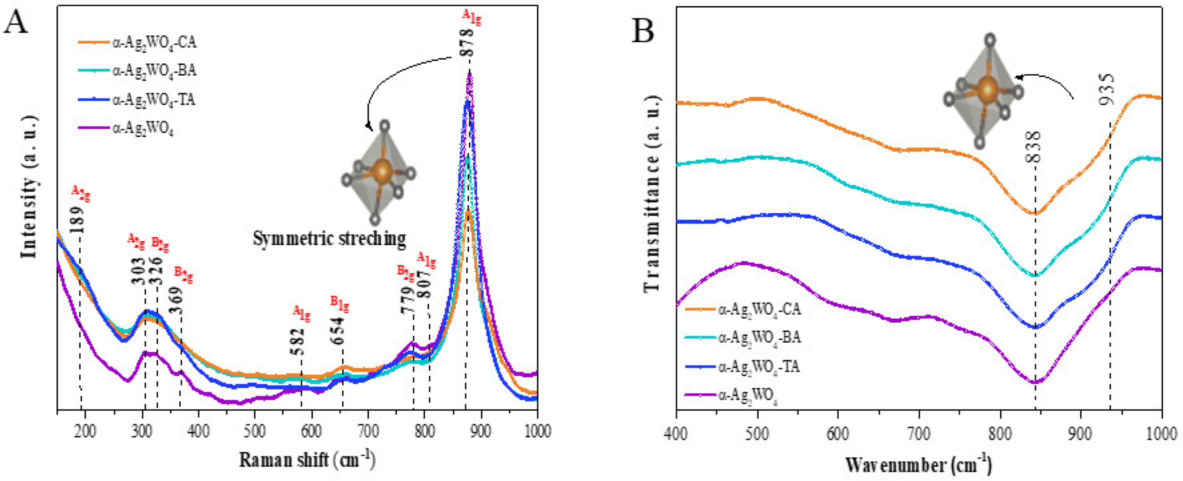

Figure S3. Raman spectroscopy of samples. The vertical dashed lines indicate the position of the Raman peaks and active modes **(A)**. FTIR spectra of samples. The vertical lines indicate the relative positions of the infrared-active modes **(B)**.

SM-5 XPS: The survey spectra of the α-Ag_2WO_4 samples

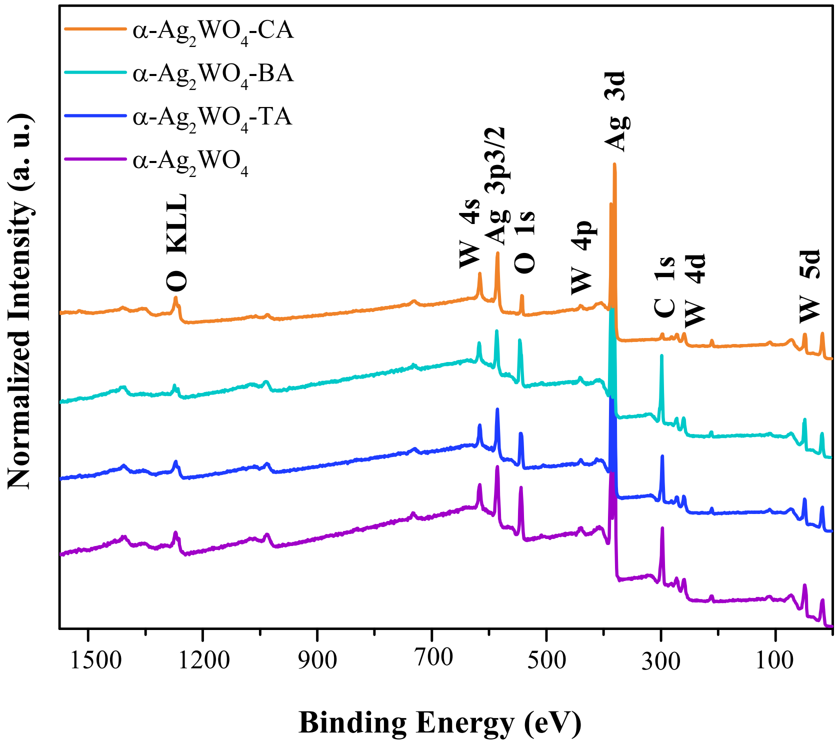

Figure S4. XPS spectra of the samples of α-Ag_2WO_4, α-Ag_2WO_4-TA, α-Ag_2WO_4-BA, and α-Ag_2WO_4-CA.

The XPS spectra of the Ag species of the α-Ag_2WO_4 samples show two bands located between ~368 and ~374 eV, which can be attributed to the binding energies of Ag 3*d*5/2 and 3*d*3/2, respectively. **Figure S5(A-B)** shows that the Ag 3*d* spectra were better fitted into two separate components for the α-Ag_2WO_4 and α-Ag_2WO_4-TA samples, which means that different Ag oxidation states are present in these samples. High intensity peaks, approximately around 368 eV and 374 eV related to the $3d_{5/2}$ and $3d_{3/2}$ orbitals, respectively, are related to the binding energy of Ag^+. The lower intensity peaks related to the $3d_{5/2}$ and $3d_{3/2}$ orbitals, obtained by fit, demonstrate the presence of Ag in the 0-oxidation state. In **Figure S5(C)** it shows that the Ag 3*d* spectra were better fitted using only one component around 367 eV and 374 eV related to the $3d_{5/2}$ and $3d_{3/2}$ orbitals, respectively, are related to the binding energy of Ag^+, and reveal the presence of only Ag^+ in the α-Ag_2WO_4-BA sample. **Figure S5(D)** shows that the Ag 3*d* spectra were best fitted using two components around 368 eV related to $3d_{5/2}$ orbitals, and only one component around 374 related to $3d_{3/2}$ orbitals. The results indicate that there is a small amount of Ag^0 in the sample.

The XPS spectra of the W species of α-Ag_2WO_4 are illustrated in **Figure S5(E-H).** The spectra show two bands located between ~36 and ~34 eV, which can be attributed to W $4f_{7/2}$ and $4f_{5/2}$ binding energies, respectively, and a broad peak related to W $5p_{3/2}$ located between 40.7 and 41.2 eV. The deconvoluted peak of W 4*f* can be adjusted into two peaks, corresponding to the typical binding energies of oxidation state W^{6+} (centered at 37.0 and 34.8 eV). Additionally, other peaks can also be observed, corresponding to the typical binding energies of W^{5+} (centered at 37.6 and 34.8 eV).

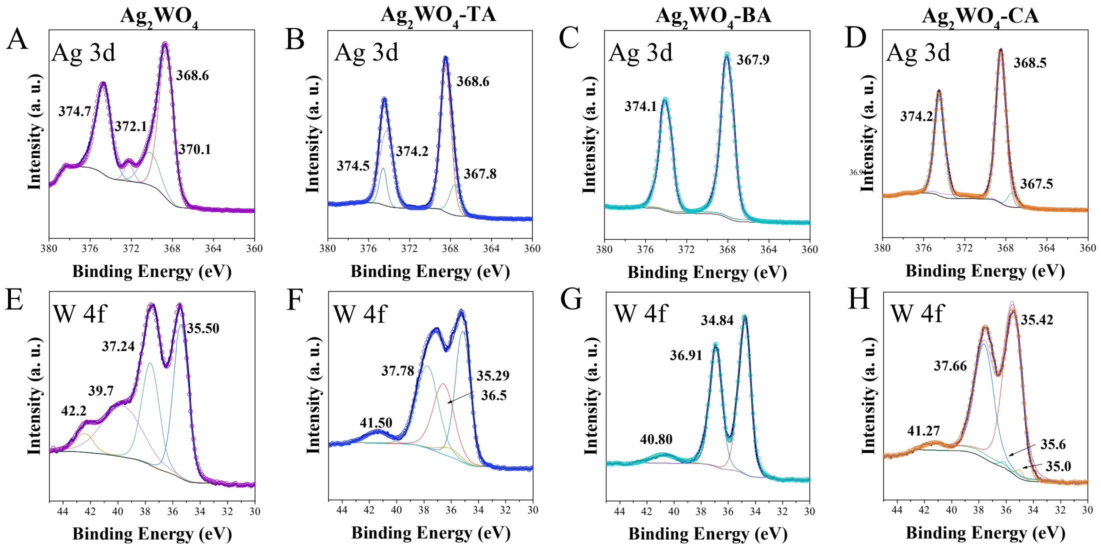

Figure S5. High-resolution XPS spectra of Ag 3d **(A, B, C, and D)** and W 4f **(E, F, G, and H)** orbitals.

SM-6 UV-vis analysis of the α-Ag_2WO_4 samples

The results of the UV-Vis diffuse reflectance spectra of the α-Ag_2WO_4 samples are shown in **Figure S6(A-D)**.

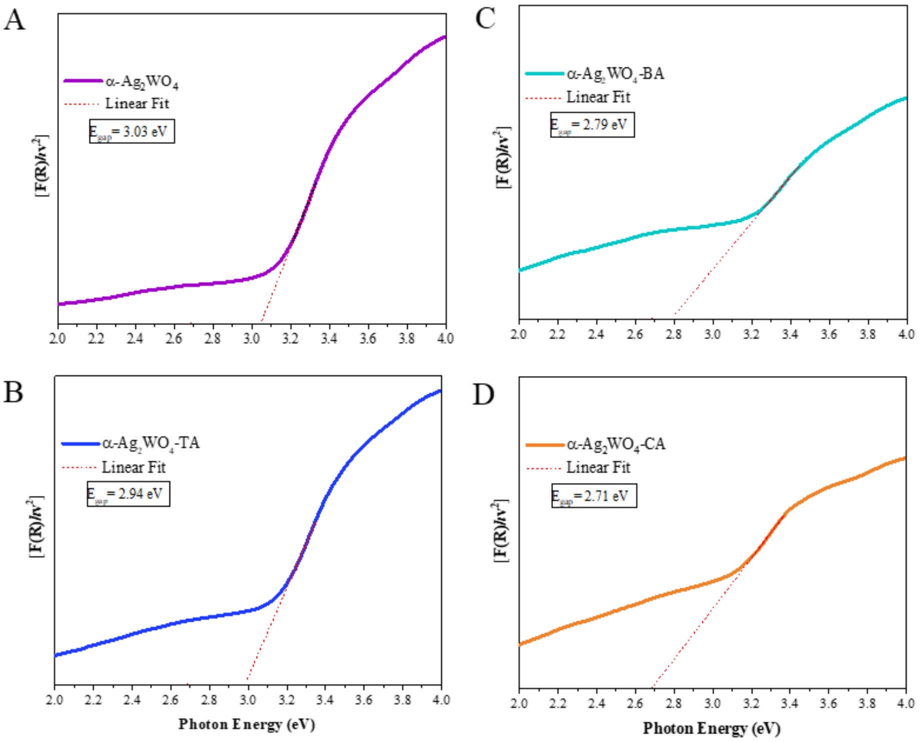

Figure S6. Determination of the E_{gap} values. Plots of transformed Kubelka-Munk function versus photon energy for α-Ag_2WO_4 **(A)**, α-Ag_2WO_4-TA **(B)**, α-Ag_2WO_4-BA **(C),** and α-Ag_2WO_4-CA **(D)** samples.

The α-Ag_2WO_4-based materials exhibit an optical absorption spectrum governed by direct electronic transitions between the valence and the conduction bands. The obtained E_{gap} values were 3.03, 2.94, 2.79, and 2.71 eV, for α-Ag_2WO_4, α-Ag_2WO_4-TA, α-Ag_2WO_4-BA, and α-Ag_2WO_4-CA, respectively.

SM-7 Photocatalytic activity

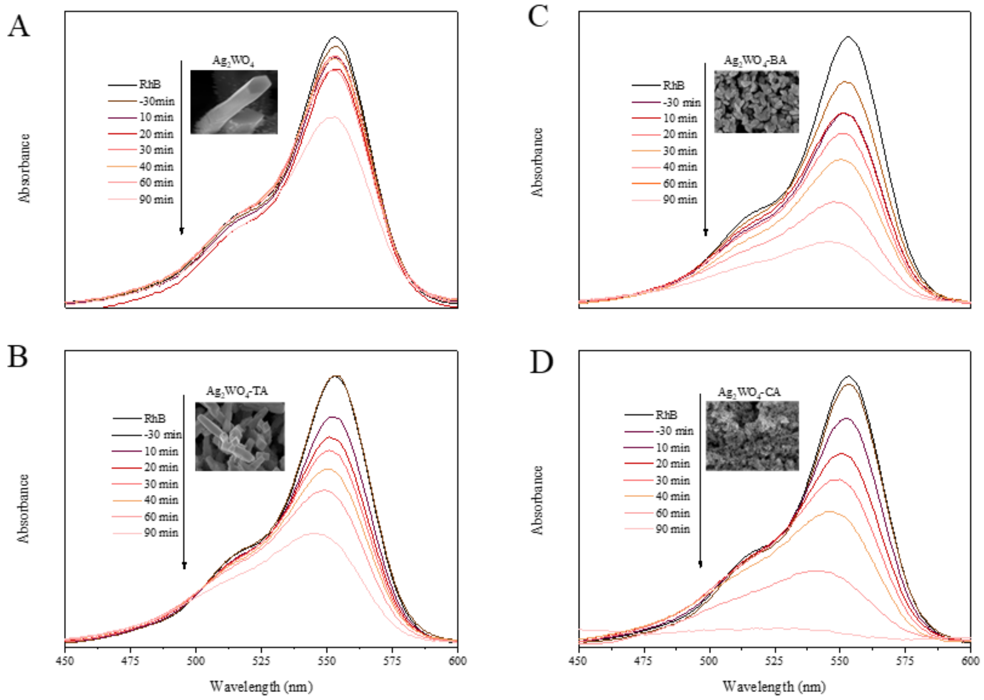

Figure S7. Evolution of UV–vis absorption spectra after 90 min of illumination for the degradation of RhB by the α-Ag_2WO_4 **(A)**, α-Ag_2WO_4-TA **(B)**, α-Ag_2WO_4-BA **(C)**, and α-Ag_2WO_4-CA **(D)** samples.

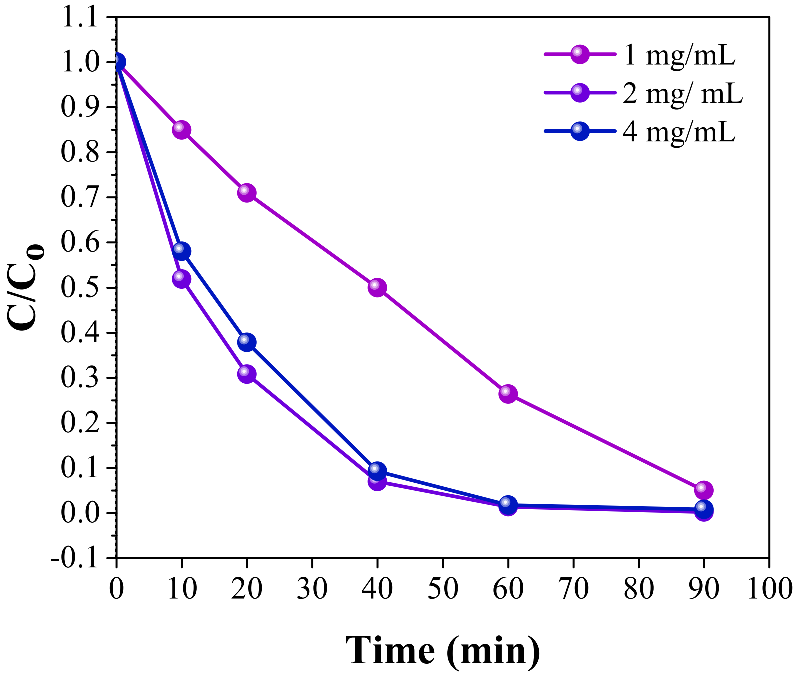

Figure S8. Photocatalytic RhB degradation profiles using the α-Ag_2WO_4-CA at different concentrations.

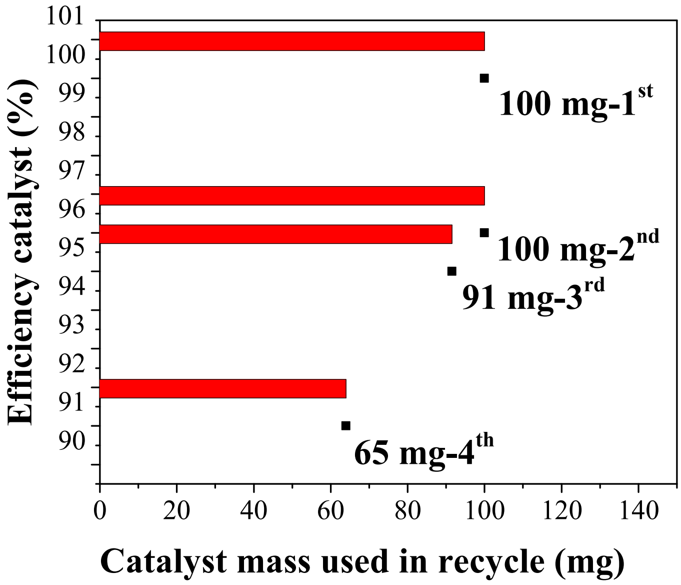

Figure S9. Relationship between loss of mass of the α-Ag_2WO_4-CA and degradation efficiency during the catalytic cycles.
